# A Bifunctional Liquid Fuel Cell Coupling Power Generation and V^3.5+^ Electrolytes Production for All Vanadium Flow Batteries

**DOI:** 10.1002/advs.202207728

**Published:** 2023-04-20

**Authors:** Shibo Sun, Liwei Fang, Hui Guo, Liping Sun, Yong Liu, Yuanhui Cheng

**Affiliations:** ^1^ State Key Laboratory of Organic‐Inorganic Composites College of Chemical Engineering Beijing University of Chemical Technology Beijing 100029 P. R. China; ^2^ China Energy Technology and Economics Research Institute China Energy Investment Corporation Ltd. Beijing 102211 P. R. China

**Keywords:** electrochemistry, energy storage materials, flow batteries, fuel cells

## Abstract

All vanadium flow batteries (VFBs) are considered one of the most promising large‐scale energy storage technology, but restricts by the high manufacturing cost of V^3.5+^ electrolytes using the current electrolysis method. Here, a bifunctional liquid fuel cell is designed and proposed to produce V^3.5+^ electrolytes and generate power energy by using formic acid as fuels and V^4+^ as oxidants. Compared with the traditional electrolysis method, this method not only does not consume additional electric energy, but also can output electric energy. Therefore, the process cost of producing V^3.5+^ electrolytes is reduced by 16.3%. This fuel cell has a maximum power of 0.276 mW cm^−2^ at an operating current of 1.75 mA cm^−2^. Ultraviolet–visible spectrum and potentiometric titration identify the oxidation state of prepared vanadium electrolytes is 3.48 ± 0.06, close to the ideal 3.5. VFBs with prepared V^3.5+^ electrolytes deliver similar energy conversion efficiency and superior capacity retention to that with commercial V^3.5+^ electrolytes. This work proposes a simple and practical strategy to prepare V^3.5+^ electrolytes.

## Graphical abstract

A novel concept for preparing vanadium electrolyte coupled with electric power generation is proposed to reduce the production cost of vanadium electrolyte. This bifunctional liquid fuel cell has a maximum power of 0.276 mW cm^−2^ at the operating current of 1.75 mA cm^−2^. The process cost of producing V^3.5+^ electrolytes is reduced by 16.3%.

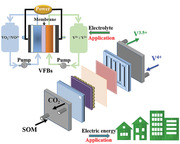

## 1 Introduction

The rapid demand for renewable energy, such as solar and wind power, has driven the development of grid‐scale long‐duration energy storage technologies.^[^ ^1^ ^]^ Aqueous flow batteries (AFBs) have attracted extensive attention in the scientific and industrial communities due to their advantages of modularity, independence of power and energy, tolerance of deep charge and discharge, and enhanced safety.^[^ ^2^ ^]^ Various AFB systems have been proposed and demonstrated in this decade, including all vanadium flow batteries (VFBs),^[^ ^3^ ^]^ zinc‐iron flow batteries,^[^ ^4^ ^]^ zinc‐bromine flow batteries,^[^ ^5^ ^]^ chromium‐iron flow batteries,^[^ ^6^ ^]^ zinc‐nickel flow batteries,^[^ ^7^ ^]^ zinc‐air flow batteries,^[^ ^8^ ^]^ and so on. Among them, the most‐developed system today is the all VFBs. VFBs not only include the general advantages of AFBs but also eliminate the issue of electrolyte pollution caused due to ion cross by using the same vanadium ions for both anode and cathode.^[^ ^9^ ^]^ Hundreds of VFB energy storage power stations have been built in a wide power range from kW to 1000 MW and a broad storage time of 0.5–20 h.^[^ ^10^ ^]^ Therefore, VFB is considered one of the most suitable technologies for long‐term energy storage.

However, the capital cost of VFBs ($400–500 kWh^−1^) remains too high for widespread commercial application.^[^ ^11^ ^]^ As a critical component of VFBs, the cost of electrolytes accounts for a large part of the entire battery system. The cost of commercial vanadium electrolyte is $118 kWh^−1^, about 31% of the total cost of a 10 kW/120 kWh VFB system.^[^ ^12^ ^]^ Furthermore, the portion of electrolyte cost in the entire VFB cost increases to 43% of the total cost of a 10 MW/40 MWh VFB system.^[^ ^13^ ^]^ The high price of electrolytes hinders the commercial application of VFBs. Therefore, reducing the cost of electrolytes has become a significant challenge for VFBs. The V^3.5+^ electrolyte is the product of mixing V^4+^ and V^3+^ in equal proportion. Commercially, V^3.5+^ is mainly used as the initial electrolyte of both the anode and cathode sides of VFBs in the industry because of easy storage, transportation, and no initial re‐balancing the anode and cathode capacities.^[^ ^14^ ^]^ After the first full charge, V^3.5+^ was converted into V^2+^ at the anode side and changed into V^5+^ at the cathode side, which are the ideal charge state of the anode and cathode chemicals, respectively.^[^ ^9b^ ^]^ Currently, the production of V^3.5+^ electrolyte mainly uses V_2_O_5_ with relatively low cost as the raw vanadium compared with other vanadium precursors. The traditional route for producing V^3.5+^ contains the first reduction of V^5+^ to V^4+^ and the following reduction of V^4+^ to V^3+^.^[^ ^15^ ^]^ The reduction of V^5+^ to V^4+^ can easily be achieved using a residue‐free organic reduction method with reducing agents such as oxalic acid.^[^ ^16^ ^]^ However, the process of V^4+^ to V^3+^ with reducing agents is exceptionally sluggish, which is the critical challenge for preparing V^3.5+^ electrolytes. Currently, electrochemical reduction of V^4+^ to V^3+^ has been employed to replace chemical reduction through electrolysis by coupling with either the oxidation of V^4+^ to V^4.5+^ or oxygen evolution at another side.^[^ ^17^ ^]^ Unfortunately, the former requires an additional reduction process for the surplus V^4.5+^, while the latter causes the corrosion of anodes.^[^ ^18^ ^]^ In addition, these two methods both consume electric energy.

Here, a novel concept for preparing vanadium electrolytes coupled with electric power generation has been proposed to reduce the production cost of vanadium electrolytes. A bifunctional liquid fuel cell was constructed by small organic molecules (SOMs) as fuels at the anode side and V^4+^ as oxidants at the cathode side. SOMs decompose to water and carbon dioxide without other impurities. To find a rational guideline for selecting the best SOM, the catalytic oxidation reactions of formic acid, methanol, and oxalic acid were studied from both the thermodynamics and dynamics views. Formic acid was selected as the best reducing agent. A prototype fuel cell employing formic acid as fuels and V^4+^ ions as oxidants was designed. This fuel cell has an open circuit voltage of 0.38 V and a maximum power of 0.276 mW cm^−2^. The V^3.5+^ electrolyte was prepared, and additional electric energy was released by controlling the discharge time. The valence of the self‐made vanadium electrolytes is 3.48 ± 0.06 during four continuous batches, close to the ideal form of 3.5, and no impurities are produced. Significantly, the self‐made electrolyte has similar efficiency and superior capacity retention rate to that of commercial electrolytes. This strategy opens a new way to prepare vanadium electrolytes and reduces the production cost.

## 2 Results and Discussion

### 2.1 Mechanism of Bifunctional Liquid Fuel Cells

**Figure 1:**
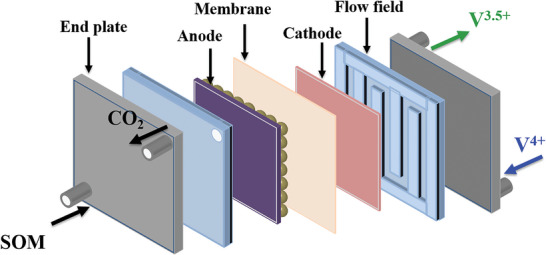
The diagram and reaction mechanism of a bifunctional liquid fuel cell.

**Figure 2:**
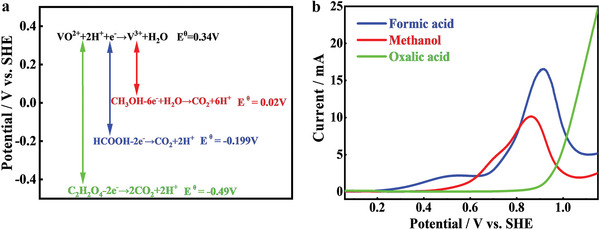
a) Standard redox potential of V^4+^/V^3+^, CH_3_OH, HCOOH, and C_2_H_2_O_4_; b) LSV curves of CH_3_OH, HCOOH, and C_2_H_2_O_4_ on Pt/C catalyst.

The reaction mechanism and processes of this novel liquid fuel cell are shown in **Figure** **1**. Small organic molecules (SOMs) are selected as fuels and oxidized to carbon oxides and waters, while V^4+^ ions are employed as oxidants and reduced to V^3.5+^ by controlling the output power energy. The open‐circuit voltage for a typical fuel cell is determined by the standard redox potentials of fuel oxidation and oxidants reduction. The standard redox potential of V^4+^ to V^3+^ is 0.34 V versus standard hydrogen electrode (SHE). For an ideal liquid fuel, SOM should have a lower redox potential than V^4+^/V^3+^, impurity‐free and low cost. Considering their requirements, formic acid, methanol, and oxalic acid were screened to construct this bifunctional fuel cell due to their low standard redox potentials of −0.199, 0.02, and −0.49 V versus SHE.^[^ ^19^ ^]^ Moreover, their oxidation products are water and carbon dioxide. Accordingly, the theoretical open‐circuit voltage obtained from the standard redox potential differences between SOM fuels and V^4+^ oxidants decrease in the below order, oxalic acid (0.83 V) > formic acid (0.54 V) > methanol (0.32 V) as displayed in **Figure** **2**a. Oxalic acid is thermodynamically more unfavorable than formic acid and methanol.

To find a rational guideline for selecting the best SOM, the catalytic oxidation reactions of formic acid, methanol, and oxalic acid were studied by linear sweep voltammetry (LSV) in the typical three‐electrode system. Commercial Pt/C with 40wt% loading was used as catalysts to facilitate the oxidation of SOMs. LSV curves in Figure 2b indicate that the onset oxidation potential of the three SOMs was in the order, oxalic acid (0.80 V vs. SHE)> methanol (0.39 V vs. SHE) > formic acid (0.17 V vs. SHE). Furthermore, formic acid has a larger oxidation current than other SOMs, indicating that formic acid has the best catalytic activity. This is different from the standard redox potential order of the three SOMs only considering thermodynamic driving force. Previous studies have shown that the direct and indirect reaction pathways of methanol are controlled by initial C—H and O—H bond activation, respectively. The primary path requires an integrated size of 3–4 Pt atoms, while the secondary path is much less sensitive. The CO that forms inhibits the surface at potentials below 0.66 V versus SHE. Therefore, the oxidation pathway of methanol on Pt/C is more complex than that of formic acid, resulting in a higher onset oxidation potential.^[^ ^20^ ^]^ Under the same conditions, the C—C bond of oxalic acid is relatively stable. Although Pt/C has a certain catalytic effect, the onset oxidation potential of oxalic acid is still too high.^[^ ^21^ ^]^ Therefore, the catalytic activity of Pt/C on the three SOMs was in the order of HCOOH > CH_3_OH > C_2_H_2_O_4_. Based on the above analysis, formic acid was selected as the best reducing agent. The Tafel slope is 128 mV dec^−1^ as displayed in Figure S1 (Supporting Information). LSV curves of V^4+^ to V^3+^ were also shown in Figure S2 (Supporting Information) to verify the possibility to construct a fuel cell.

### 2.2 Production of V^3.5+^ Electrolytes

**Figure 3:**
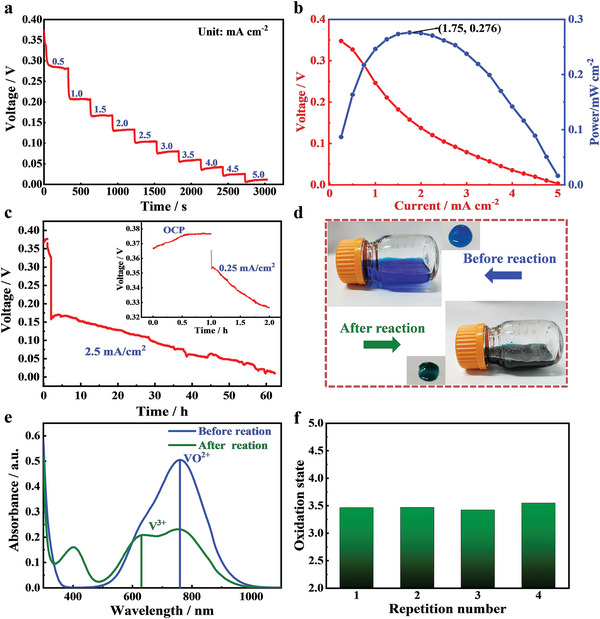
a) Discharge voltage curve of this bifunctional liquid fuel cell at different current densities, b) polarization curve and power‐density, c) discharge voltage curve at 2.5 mA cm^−2^, d) visual comparison of electrolyte before and after reaction, e) ultraviolet–visible (UV–vis) spectra of the electrolytes before and after reaction, and f) oxidation states of the electrolytes from the four repeated products.

A prototype fuel cell employing formic acid as fuels and V^4+^ ions as oxidants was designed and constructed to demonstrate the bifunctional liquid fuel cell for power generation and V^3.5+^ electrolyte production. Pt/C catalyst and carbon felt served as the anode and cathode catalysts, respectively. The discharge voltage curves were tested under different current densities. **Figure** **3**a shows that the open circuit voltage of the fuel cell is about 0.38 V. The discharge voltage presents a ladder shape with the current increase in a current increment of 0.5 mA cm^−2^, indicating that this fuel cell has good voltage stability under different currents. The discharge voltages are 0.28 V at 0.5 mA cm^−2^, 0.21 V at 1.0 mA cm^−2^, 0.13 V at 2.0 mA cm^−2^, and 0.10 V at 2.5 mA cm^−2^, respectively. In order to further determine the optimal conditions for the preparation of vanadium electrolyte, the polarization curve is displayed in Figure 3b. This fuel cell has a maximum power of 0.276 mW cm^−2^ at an operating current of 1.75 mA cm^−2^. Although the power density of the battery is low, the preparation of electrolyte does not require additional electrical energy. On the contrary, the battery can output electrical energy. V^3.5+^ electrolyte was produced through this bifunctional liquid fuel cell under the current of 2.5 mA cm^−2^ in Figure 3c. The reduction amount of V^4+^ ions can be controlled by regulating the discharge time or capacity of this fuel cell, so as to prepare V^3.5+^ electrolyte. During the battery discharge process, the discharge voltage will gradually decrease with the discharge process. This is because the concentration of the reactants (formic acid and V^4+^ ions) gradually decrease, resulting in a significant increase in concentration polarization. Comparing the electrolyte at the cathode side before and after the reaction by visual observation, the color of the V^4+^ initial solution was blue before the reaction, and the color of the electrolyte changed to dark green after the reaction, indicating that the valence state of the vanadium ion had reduced (Figure 3d). The valence state of the electrolyte before and after the reaction was tested by the ultraviolet–visible (UV–vis) spectrum. There was only one characteristic absorption peak at 760 nm in the initial solution, while an additional absorption peak 610 nm appeared after the reaction (Figure 3e). Previous studies have identified that the characteristic absorption peaks near 610 and 760 nm belong to V^3+^ and V^4+^, respectively.^[^ ^22^ ^]^ Therefore, V^3+^ was produced in the solution in this novel liquid fuel cell. The valence state of the electrolyte was further characterized by potentiometric titration based on the oxidizing–reduction principle (Figure 3f). Four batches experiments were conducted to evaluate the feasibility of large‐scale continuous electrolyte production. In order to verify the activity and stability of platinum catalyst under this condition, LSV test was carried out on the catalyst before and after the reaction (Figure S3, Supporting Information). It can be seen that after 250 hours of reaction, the initial oxidation potential of formic acid did not change significantly, and the current peak value decreased slightly, further proving the feasibility of continuous production. The valence state of four batches of continuously prepared vanadium electrolytes is +3.48 ± 0.06, which is very close to the ideal state of +3.5. This indicates that this novel liquid fuel cell successfully prepared V^3.5+^ electrolytes. Compared with the traditional electrolysis method, this method not only does not consume additional electric energy, but also can output electric energy. Therefore, the process cost of producing V^3.5+^ electrolytes is reduced by 16.3% as evaluated in Tables S1–S3 (Supporting Information). We further calculated the production cost for a preparing system with a production capacity of 2000 m^3^ (100 MWh) per year. The bifunctional liquid fuel cell stacks were constructed by 50 sections in series with an effective area of 0.6 m^2^ per section. Eight stacks are enough to realize 2000 m^3^ electrolytes per year. The detailed items of size, quantity, and cost for core components of one stack with a production capacity of 30 L h^−1^ are listed in Table S4 (Supporting Information). The detailed cost for consumed raw material and energy are calculated in Table S5 (Supporting Information), which reduces 14% compared with the traditional method cost listed in Table S6 (Supporting Information). In addition, we compared the solution color at the formic acid side before and after the discharge of the bifunctional fuel cell. The solution changes from initial colorless to light‐blue after the reaction. ICP measurement shows the content of vanadium ions in formic acid side solution is only 3.21 g L^−1^, accounting for only 4.2% of total vanadium ions.

### 2.3 VFB Performance

**Figure 4:**
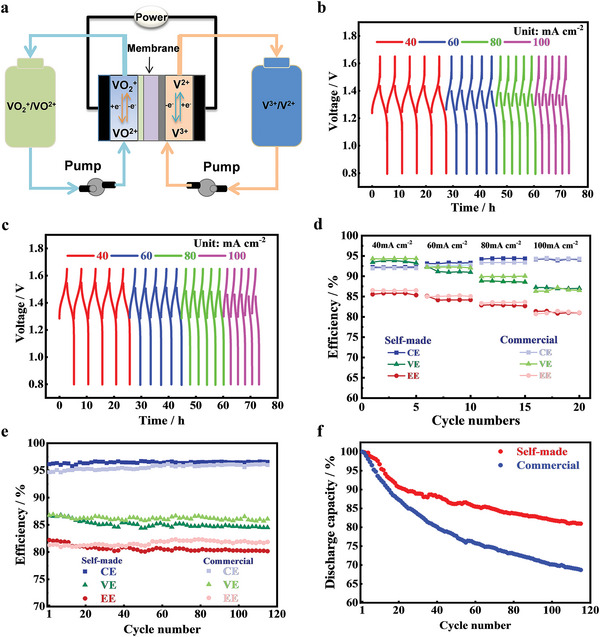
a) Schematic diagram of a VFB, charge and discharge voltage curves at different current densities using b) self‐made electrolyte and c) commercial electrolyte, d) current efficiency (CE), voltage efficiency (VE), and energy efficiency (EE) of VFBs with self‐made and commercial electrolyte at different currents, e) efficiency plots, and f) discharge capacity retention curves at 100 mA cm^−2^ using self‐made and commercial vanadium electrolytes.

The quality of prepared V^3.5+^ electrolytes by the bifunctional fuel cells was investigated by evaluating the electrochemical performance of VFBs. It was used as both the anode and cathode electrolytes for VFBs (**Figure** **4**a). Commercial V^3.5+^ electrolytes were also calculated as the benchmark. The charge–discharge tests of VFBs were conducted at 40, 60, 80, and 100 mA cm^−2^ as shown in Figure 4b and Figure S4a (Supporting Information). As the current density increases from 40 to 100 mA cm^−2^, the average charge voltage of VFBs with prepared V^3.5+^ electrolyte increases from 1.38 to 1.43 V, and the average discharge voltage decreases from 1.29 to 1.23 V. The prepared V^3.5+^ electrolytes and commercial V^3.5+^ electrolytes have close average charge and discharge voltage values (Figure 4c and Figure S4b, Supporting Information). Coulomb efficiency (CE), voltage efficiency (VE), and energy efficiency (EE) were compared between VFBs with prepared V^3.5+^ electrolytes and VFBs with commercial electrolytes under different current densities. They have very close CE, VE, and EE to each other under different current densities. For example, CE, VE, and EE of VFBs with commercial vanadium electrolytes are 94.3%, 86.5%, and 80.7% under 100 mA cm^−2^, while CE, VE, and EE of VFBs with self‐made vanadium electrolyte are 94.1%, 87.1%, and 81.2%, respectively (Figure 4d). Stability tests were conducted at 100 mA cm^−2^ to further verify the stability of the prepared electrolyte. After 120 charge–discharge cycles, CE, VE, and EE of VFBs with self‐made electrolytes are 96.5%, 84.5%, and 80.2%, and the reduction of efficiency is negligible. The CE, VE, and EE of VFBs with commercial electrolytes are 96.2%, 86.1%, and 81.9% (Figure 4e). The two kinds of electrolytes have similar efficiency. Notably, the discharge capacity retention rate of the prepared vanadium electrolytes is 81% after 115 cycles, and the decay rate is only 0.165% cycle^−1^ (Figure 4f and Figure S5a, Supporting Information). However, the discharge capacity retention rate of commercial vanadium electrolyte was only 68.4% after 115 cycles, and the decay rate reached 0.272% cycle^−1^ (Figure 4f and Figure S5b, Supporting Information). We have compared the performance parameters of prepared vanadium electrolytes with recently published literatures as displayed in Table S7 (Supporting Information). The vanadium electrolytes prepared by this work is superior to most vanadium electrolytes in terms of energy conversion efficiency and discharge capacity retention. Therefore, compared with commercial V^3.5+^ electrolytes, the prepared V^3.5+^ electrolytes have similar energy conversion efficiency, but better stability and capacity retention rate.

## 3 Conclusion

In summary, a novel method of preparing V^3.5+^ electrolytes for VFBs using a bifunctional liquid fuel cell was demonstrated in this report. Candidates for anodic fuels were selected based on logical guidelines. Formic acid delivers the best candidates due to its fast reaction rate, while methanol and oxalic acid exhibit sluggish kinetics. A prototype bifunctional fuel cell employing formic acid as fuels and V^4+^ ions as oxidants was designed and constructed to generate power generation and prepare V^3.5+^ electrolytes. The reduction amount of V^4+^ ions can be controlled by regulating the discharge time or capacity of this fuel cell, so as to prepare V^3.5+^ electrolytes. The valence state of four batches of continuous prepared vanadium electrolyte is 3.48 ± 0.06, which is close to the ideal form of +3.5, and no impurities are produced. Significantly, VFBs with self‐made vanadium electrolyte have similar energy conversion efficiency and superior capacity retention rate to commercial vanadium electrolytes. The decay rate of discharge capacity is reduced to 0.165% cycle^−1^. This dual‐function system of fuel cells and electrosynthesis opens a new way to prepare V^3.5+^ electrolytes.

## 4 Experimental Section

### Chemicals

VOSO_4_•5H_2_O (99%) was purchased from Shenyang Haizhongtian Fine Chemical Factory. Methanol (AR, ≥99.5%) and ethanol (AR, ≥99.5%) were purchased from Beijing Tongguang Fine Chemical Co., Ltd. Formic acid (≥88%) was purchased from Fuchen (Tianjin) Chemical Reagent Co., Ltd. Oxalic acid (AR, 99%) was purchased from Aladdin. Carbon paper and Pt/C catalyst were obtained from Shanghai Hesen Electrical Co., Ltd. The carbon felt was purchased from Henan Chuanyu New Material Technology Co., Ltd. with a thickness of 6 mm. The carbon fiber type is T700. All chemicals were used without further purification.

### Electrochemical Reaction Analysis of Small Organic Molecules

The electrochemical test of different SOMs was carried out with a three‐electrode electrochemical cell. The three‐electrode system consisted of a glassy carbon electrode (0.196 cm^2^) coated with Pt/C catalyst as the working electrode, a carbon rod as the counter electrode, and a Hg/Hg_2_SO_4_ electrode as the reference electrode. And the reference electrode was placed in a salt bridge filled with saturated K_2_SO_4_ solution. The electrolytes were 0.5 mol L^−1^ HCOOH + 0.5 mol L^−1^ H_2_SO_4_, 0.5 mol L^−1^ CH_3_OH + 0.5 mol L^−1^ H_2_SO_4_, and 0.5 mol L^−1^ C_2_H_2_O_4_ + 0.5 mol L^−1^ H_2_SO_4_, respectively. The 5.0 mg Pt/C catalyst was dissolved in a mixture of 50 µL Nafion solution (5 wt%) and 950 µL anhydrous ethanol, and the catalyst ink was obtained after ultrasonic dispersion. 10 µL ink was dropped onto the glassy carbon electrode and dried at room temperature for electrochemical tests. The LSV test of SOMs was performed at a voltage of −0.4 to 0.5 V with a sweep speed of 5 mV s^−1^. The test of V^4+^ electrolyte needs to replace the working electrode, electrolyte, and working conditions: the glassy carbon electrode was replaced with the electrode clamp with carbon felt, the electrolyte uses the mixed solution of 1.5 m VOSO_4_ and 3 m H_2_SO_4_, and the test voltage range of cyclic voltammetry (CV) and LSV was changed to −0.4 to 0.1 V. All electrochemical tests were performed on a CHI760E electrochemical instrument (Shanghai Chenhua, China).

### Demonstration of Bifunctional Fuel Cells

Carbon felt was used as the cathodic electrode and the electrolyte was a mixture of 1.5 mol L^−1^ VOSO_4_ and 3 mol L^−1^ H_2_SO_4_. Carbon paper with a gas diffusion layer (GDL) was used as the anodic electrode and the electrolyte was a mixture of 6 mol L^−1^ HCOOH and 3 mol L^−1^ H_2_SO_4_. Nafion115 membrane was used as the ion exchange membrane (Nafion115, DuPont). The membrane electrode assembly (MEA, 3 × 3 cm^2^) was prepared by spraying the catalyst directly on carbon paper. The 27 mg Pt/C catalyst was dissolved in 1 mL ethanol/Nafion (5 wt%) mixed solution and sprayed uniformly on the GDL side of carbon paper, resulting in a Pt/C loading of 3 mg cm^−2^. The polarization, power‐density, and discharge–voltage curves were measured under different current densities. The range of the polarization curve test and power density curve test is 0–5 mA cm^−2^, the interval is 0.25 mA cm^−2^, and the time of each current density test is 10 s. The range of the discharge voltage curve test under different current densities is 0–5 mA cm^−2^, the interval is 0.5 mA cm^−2^, and the test time of each current density is 300 s. Then, the 2.5 mA cm^−2^ was selected for discharge to prepare the electrolyte, and the volume of both positive and negative electrolytes was 70 mL. The liquid flow rate is about 85 mL min^−1^ and the utilization rate of SOM is about 70%.

### Characterization of V^3.5+^ Electrolyte

First, the change in the valence state of the vanadium electrolyte was judged by comparing the color of the vanadium electrolyte before and after the reaction. Second, ultraviolet–visible spectroscopy (UV–vis) was performed to determine the change of ion species by studying the characteristic peak of electrolytes. Previous studies have indicated the characteristic peaks of different valence vanadium ions: V (III) was 610 nm, and V (IV) was 765 nm. Finally, the accurate valence state of the vanadium electrolyte was obtained by potentiometric titration based on the oxidizing‐reduction principle. The amount of potassium permanganate consumed to complete two leaps were recorded respectively. And the accurate concentration of vanadium electrolyte was obtained by calculation.

### Performance Test of VFBs

To measure the performance of the self‐made vanadium electrolyte, a constant current charge–discharge test and cyclic stability test were carried out on the self‐made and commercial vanadium electrolyte. The battery is divided into end plate, graphite plate, carbon felt, and Nafion115 membrane from the outside to the inside. The activated electrode area is 3 × 3 cm^2^. And the volume of both positive and negative electrolytes is 30 mL. The constant current charge–discharge tests were conducted at 40, 60, 80, and 100 mA cm^−2^, respectively, and the cycle stability tests were conducted at 100 mA cm^−2^. The charge and discharge cut‐off voltage for the above test were set as 1.6 and 0.8 V. Finally, the charge–discharge curve, efficiency, discharge capacity, and discharge capacity retention rate of the battery were obtained.

## Conflict of Interest

The authors declare no conflict of interest.

## Supporting information

Supporting information

## Data Availability

The data that support the findings of this study are available from the corresponding author upon reasonable request.

## References

[1] a) G. Crabtree , Nature 2015, 526, S92; 26509952 10.1038/526S92a

[2] a) M. J. Nan , L. Qiao , Y. Q. Liu , H. M. Zhang , X. K. Ma , Prog. Chem. 2022, 34, 1402;

[3] a) S. Maria , J. Electrochem. Soc. 2022, 169, 070513;

[4] a) Z. Q. Chen , W. T. Yu , Y. F. Liu , Y. K. Zeng , Q. J. He , P. Tan , M. Ni , Chem. Eng. J. 2021, 405, 126684;

[5] a) Z. C. Xu , Q. Fan , Y. Li , J. Wang , P. D. Lund , Renewable Sustainable Energy Rev. 2020, 127, 109838; 10.1016/j.rser.2020.109883 PMC7183989 34234614

[6] a) Y. Ahn , J. Moon , S. E. Park , J. Shin , W. J. Choi , K. J. Kim , Chem. Eng. J. 2021, 421, 127855;

[7] a) Y. H. Cheng , H. Guo , Chem. Eng. Sci. 2021, 232, 116372;

[8] a) L. S. Gao , X. Gao , P. Jiang , C. Y. Zhang , H. Guo , Y. H. Cheng , Small 2022, 18, 2105892; 10.1002/smll.202105892 34898014

[9] a) R. Gundlapalli , A. Bhattarai , R. Ranjan , P. C. Ghimire , X. M. Yeo , N. A. B. Zainudin , N. Wai , F. Mahlendorf , A. Jasincuk , H. Thorsten , J. Power Sources 2022, 542, 231812;

[10] DOE Global Energy Storage Database, Distribution and scale of electro‐mechanical energy storage, 2022, https://sandia.gov/ess‐ssl/gesdb/public/projects.html (accessed: November 2022).

[11] L. Su , A. F. Badel , C. S. Cao , J. J. Hinricher , F. R. Brushett , Ind. Eng. Chem. Res. 2017, 56, 9783.

[12] C. Minke , U. Kunz , T. Turek , J. Power Sources 2017, 361, 105.

[13] a) J. Noack , L. Wietschel , N. Roznyatovskaya , K. Pinkwart , J. Tübke , Energies 2016, 9, 627;

[14] R. Woodfield , S. Glover , R. Watson , P. Nockemann , R. Stocker , J. Energy Storage 2022, 54, 105306.

[15] C. Y. Choi , S. Kim , R. Kim , Y. Choi , S. Kim , H. Jung , J. H. Yang , H. Kim , Renewable Sustainable Energy Rev. 2017, 69, 263.

[16] a) B. G. Kim , S. J. Lee , (Daejeon KR), US9406961B2 2016;

[17] M. Kazacos , I. Skyllas‐Kazacos , (VRB Power Systems, M.), US7078123B2 2006.

[18] C. Madan , L. Sharma , S. Mukerjee , A. Halder , Int. J. Hydrogen Energy 2022, 47, 22738.

[19] J. Heo , J. Y. Han , S. Kim , S. Yuk , C. Choi , R. Kim , J. H. Lee , A. Klassen , S. K. Ryi , H. T. Kim , Nat. Commun. 2019, 10, 4412. 31562304 10.1038/s41467-019-12363-7 PMC6764956

[20] a) M. Neurock , M. Janik , A. Wieckowski , Faraday Discuss. 2008, 140, 368; 10.1039/b804591g 19213327

[21] a) S. Yamazaki , Y. Yamada , N. Fujiwara , T. Ioroi , Z. Siroma , H. Senoh , K. Yasuda , J. Electroanal. Chem. 2007, 602, 96;

[22] a) N. Choi , S. Kwon , H. T. Kim , J. Electrochem. Soc. 2013, 160, A973;

