# Supplementary material for: A Bifunctional Liquid Fuel Cell Coupling Power Generation and V3.5+ Electrolytes Production for All Vanadium Flow Batteries
Source: Adv Sci (Weinh). 2023 Apr 20;10(18):2207728. doi: 10.1002/advs.202207728 (PMC10288229; doi:10.1002/advs.202207728)
Supplement: Supplementary file 1 — Supporting information [file ADVS-10-2207728-s001.pdf]

Supplementary Information

A bifunctional liquid fuel cell coupling power generation and $V^{3.5+}$ electrolytes production for all vanadium flow batteries

Shibo Sun†, Liwei Fang†, Hui Guo†, Liping Sun, Yong Liu, Yuanhui Cheng *

*Corresponding author. Email: chengyh@mail.buct.edu.cn

† These authors contributed equally to this work.

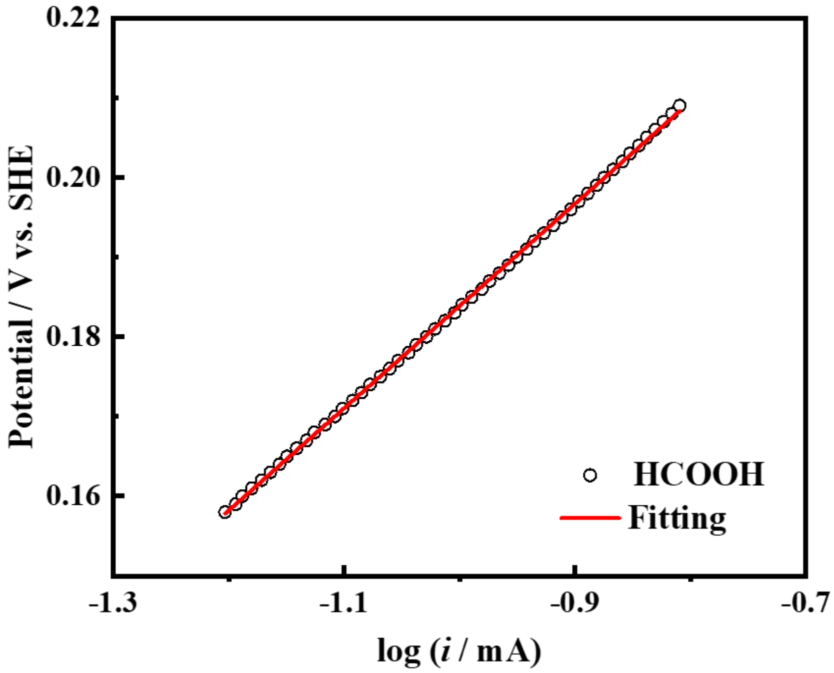

Figure S1. Tafel plots of formic acid catalyzed by Pt catalyst.

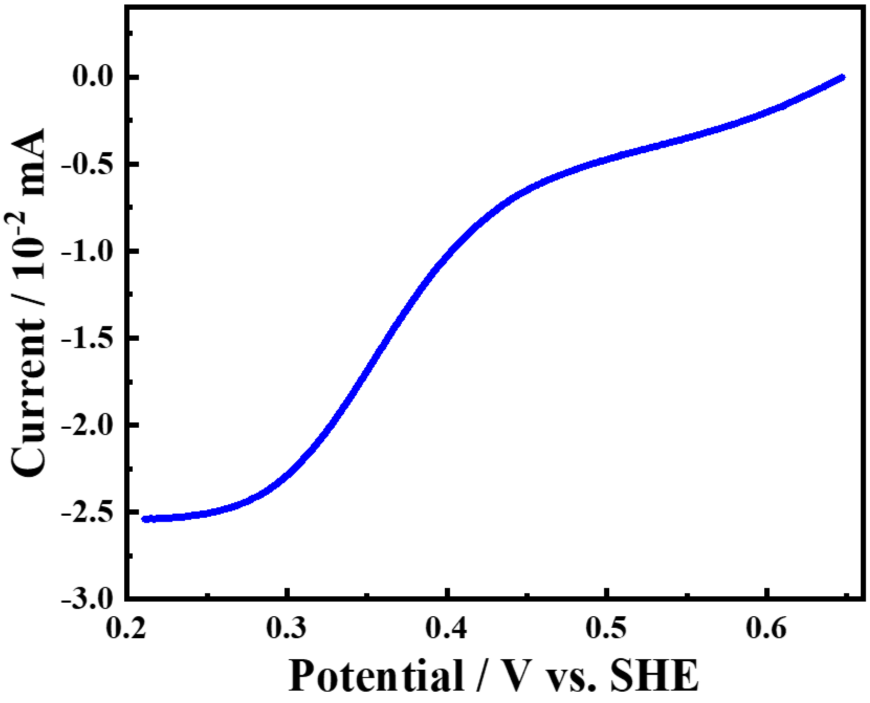

Figure S2. LSV curve of the reduction of V^{4+} to V^{3+}.

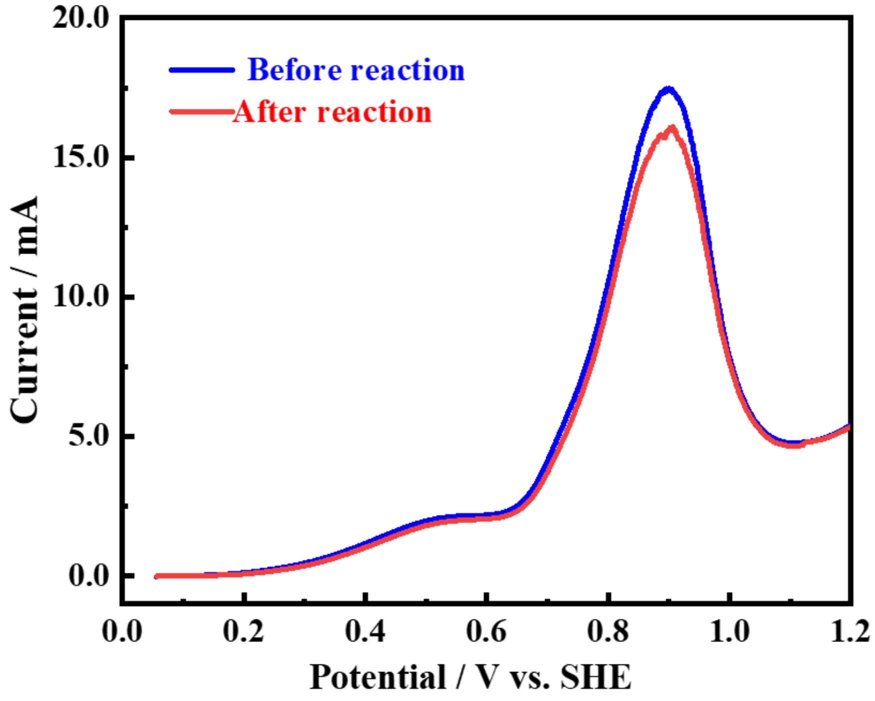

Figure S3. LVS curves of platinum electrode toward formic acid oxidation before and after long-

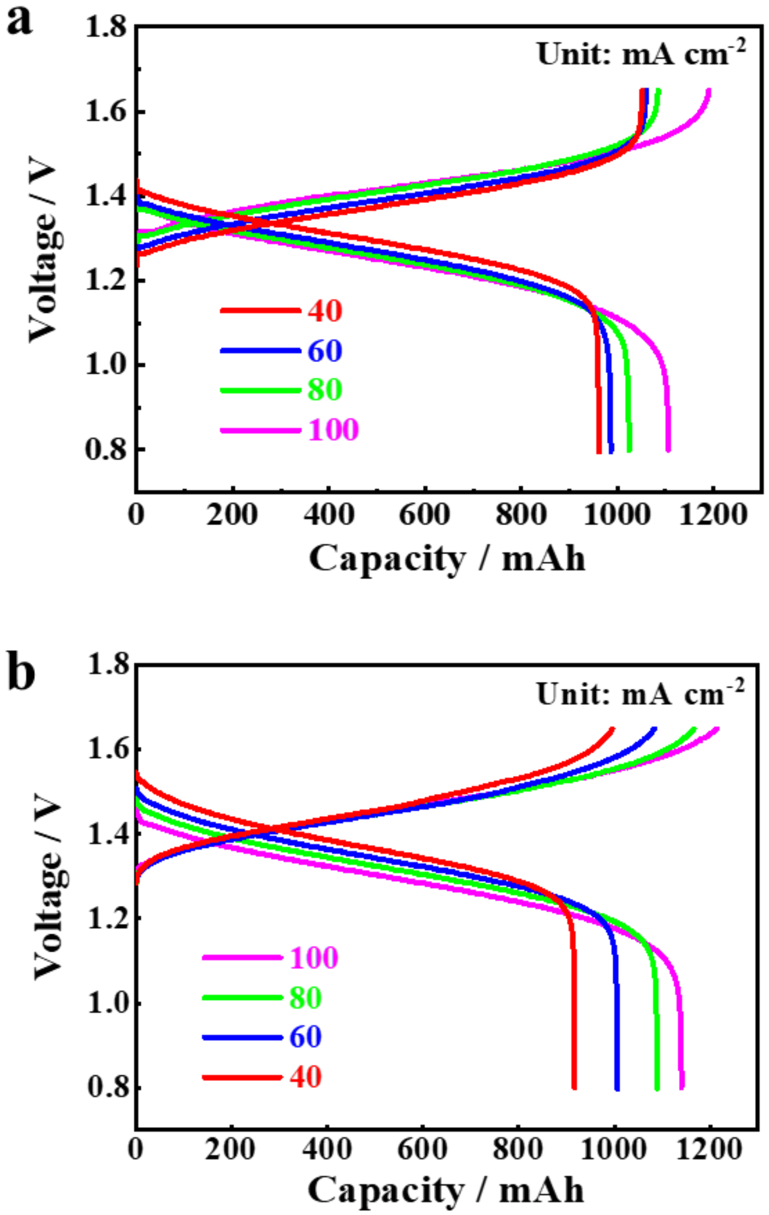

term operation.

Figure S4. Charge-discharge voltage-capacity curves of VFBs using (a) self-made vanadium electrolyte and (b) commercial vanadium electrolyte at different current densities.

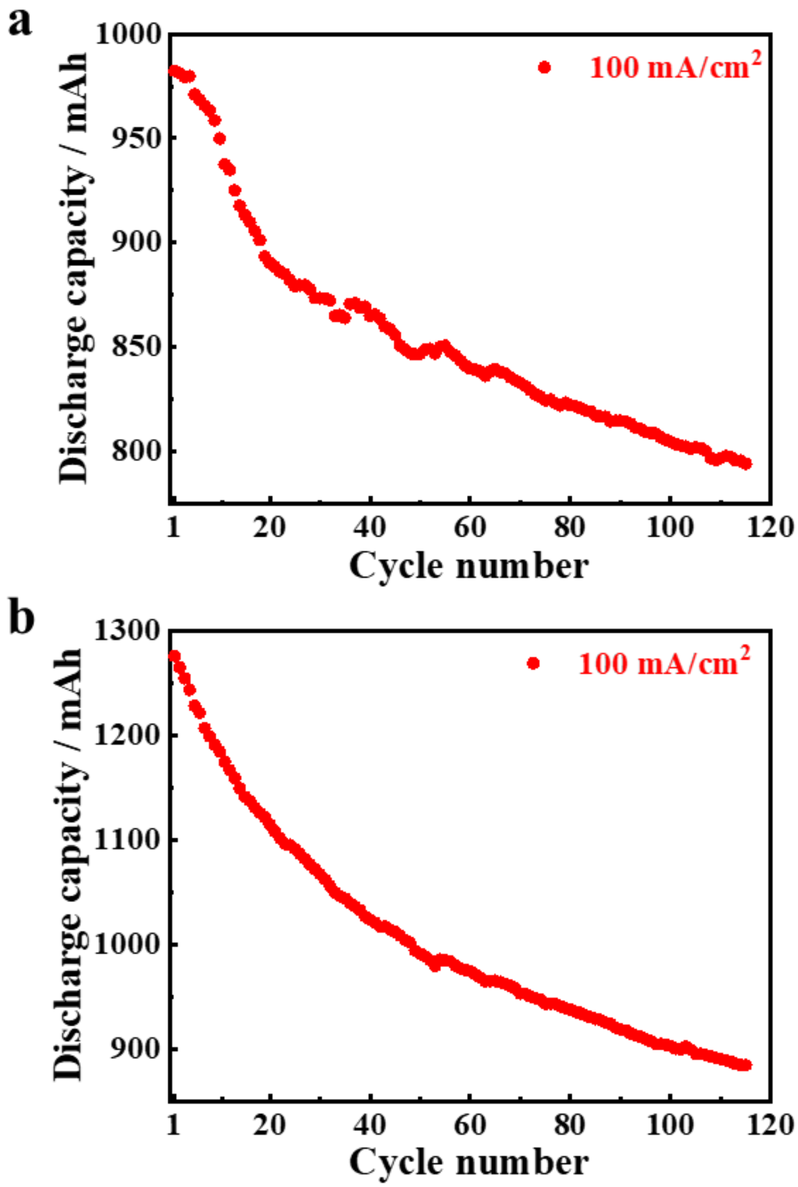

Figure S5. (a) Discharge capacity diagram of VFBs with self-made vanadium electrolyte or (b) commercial vanadium electrolyte along with cycle number at 100 mA cm^{-2}.

Table S1. Cost of the bifunctional liquid fuel cell (Production capacity: Laboratory scale).

Material	Specification	Unit	Unit cost($ k))	Usage	Cost ($)
End plate	7cm×7cm×2cm	ea	42.987 a)	2	85.974
Graphite plate	5cm×5cm×3mm	ea	7.164 b)	2	14.328
Teflon frame	5cm×5cm×3mm	ea	1.433 c)	1	1.433
Silicone gasket	5cm×5cm×0.5mm	ea	0.072 d)	5	0.36
Carbon paper	3cm×3cm	ea	1.838 e)	1	1.838
Pt/C	40wt%	g	42.987 f)	0.027	1.161
Nafion solution	5wt%	ml	1.764 g)	0.27	0.476
Ethanol	AR	L	0.792 h)	0.002	0.002
Carbon felt	3cm×3cm	cm^2	0.013 i)	1	0.013
Nafion 115 membrane	5cm×5cm	cm^2	1.86 j)	1	1.86
		Total			107.445

a) - e) Quoted from https://b2b.baidu.com.

f) Quoted from https://www.1688.com.

g) - h) Quoted from https://china.guidechem.com.

i) – j) Quoted from https://b2b.baidu.com.

k) $ stands for US dollar, ¥ stand for Chinese yuan. 1$=6.9788¥

The cost accounting of laboratory scale bifunctional liquid fuel cell was carried out, and the cost analysis was shown in Table S1. The membrane electrode assembly was mainly composed of Nafion115 membrane, carbon felt and Pt modified carbon paper. The total cost of fuel cell in laboratory scale is 107.445 US dollars, which can be compared with the cost of conventional electrolyzers in the same capacity scale.[1-3]

Table S2. Process cost for catalytic production of 1 L of $V^{3.5+}$ electrolyte (Production capacity: Laboratory scale).

Component	Requirement	Unit	Unit cost	Cost (¢ L^{-1})
Formic acid	0.375 mol	mol L^{-1} [c)]	2.328¢ mol^{-1} [d)]	0. 873
Sulfuric acid [b)]	3.0019 mol	mol L^{-1}	0. 5736¢ mol^{-1} [e)]	1.829
Generated electricity	-0.00603 kWh	kWh L^{-1}	17.0¢ kWh^{-1} [f)]	-0.103
Consumable [a)]	--	--	--	119.483
Total				122.082

a) Assumption: Electrode and membrane need to be replaced every 9000h operation. U_1 represents the total cost of electrode and membrane, including carbon paper, carbon felt, Nafion membranes, Pt/C catalysts, etc. The specific scale is shown in Table S1. t_1 represents the time it takes to produce 1L of electrolyte. U_2 represents the cost of electrodes and membranes consumed to produce 1L of electrolyte Therefore, the consumption cost of producing 1L electrolyte is $U_2 = U_1 \times t_1/9000h$.

b) Assumption: Sulfuric acid in formic acid needs to be replaced for every 100L electrolyte produced.

c) L^{-1} in every unit refers to liter of vanadium solution.

d) Quoted from https://www.100ppi.com.

e) The electricity price is calculated at 1.2 ¥ kWh^{-1}

f) ¢ stand for the cent. 1 $=100 ¢

Table S3. Process cost for conventional method of 1 L of $V^{3.5+}$ electrolyte production (Production capacity: Laboratory scale).

Component	Requirement	Unit	Unit cost	Cost (¢ L^{-1})
Reducing agent(SO_2)	0.03871	kg L^{-1} b)	50.152¢ mol^{-1} c)	1.941
Sulfuric acid	3	mol L^{-1}	0. 5736¢ mol^{-1}	1.731
Electricity consumption	0.07317	kWh L^{-1}	17.0¢ kWh^{-1}	1.244
Consumable a)	--	--	--	140.941[1-3]
		**Total**		145.857

a) Assumptions: Electrode and membrane need to be replaced every 9000h operation. U_3 represents the total cost of electrode and membrane, including carbon felt, Titanium mesh, Nafion membrane, IrO_2 catalysts, etc. The specific scale is shown in Table S1. t_2 represents the time it takes to produce 1L of electrolyte. U_4 represents the cost of electrode and membrane consumed to produce 1L of electrolyte Therefore, the consumption cost of producing 1L electrolyte is $U_4 = U_3 \times t_2/9000h$.

b) L^{-1} in every unit refers to liter of vanadium solution.

c) Quoted from http://china.makepolo.com.

The process costs of discharge preparation of electrolyte and conventional electrolysis are calculated as shown in Table S2, S3. For discharge preparation of electrolyte, the process cost for 1 L $V^{3.5+}$ electrolyte is approximately ¢122.082, while the process cost for 1 L $V^{3.5+}$ electrolyte by electrolysis preparation is approximately ¢145.857. Therefore, the process cost of electrolyte by discharge can be reduced by 16.3% compared with the electrolyte by electrolysis.

Labor costs are not included in the calculation, otherwise the gap between the two processes would be even greater. It is worth noting that the other material costs (vanadium oxysulfate) are identical between the two processes.

Table S4. Cost of one bifunctional liquid fuel cell stack (Industrial scale: 30 L/h).

Material	Specification	Unit	Unit cost ($ k))	Usage	Cost ($)
**End plate**	65cm×105cm×5cm	ea	965.782a)	2	1931.564
**Graphite plate**	62cm×102cm×10mm	ea	171.95 b)	51	8769.45
**Teflon frame**	62cm×102cm×3mm	ea	28.658 c)	50	1432.9
**Silicone gasket**	62cm×102cm×0.5mm	ea	2.636d)	152	400.672
**Carbon paper**	60cm×100cm	ea	276 e)	50	13800
**Pt/C**	40 wt%	g	42.987 f)	900	38688.3
**Nafion solution**	5 wt%	ml	1.764 g)	9000	15876
**Ethanol**	AR	L	0.792 h)	120	95.04
**Carbon felt**	60cm×100cm	ea	8.4 i)	50	420
**Nafion 115 membrane**	61cm×101cm	ea	458.378 j)	50	22918.92
		**Total**			104332.846

a) - e) Quoted from https://b2b.baidu.com.

f) Quoted from https://www.1688.com.

g) - h) Quoted from https://china.guidechem.com.

i) – j) Quoted from https://b2b.baidu.com.

k) $ stands for US dollar, ¥ stand for Chinese yuan. 1$=6.9788¥

Table S5. Process cost of this method for producing 2000 m^3 $V^{3.5+}$ electrolyte (Production capacity: Industrial scale).

Component	Requirement[c)]	Unit cost	Cost ($)
**Formic acid**	750 kmol	2328¢ $kmol^{-1}$ [d)]	17460
**Sulfuric acid** [b)]	6003.8 kmol	573.6¢ mol^{-1}	34437.796
**Generated electricity**	-12.06 MWh	170$ MWh^{-1} [e)]	-2050.2
**Consumable** [a)]	--	--	683795.038
	**Total**		733642.634

a) Assumption: Electrode and membrane need to be replaced every 9000h operation. U_1 represents the total cost of electrode and membrane, including carbon paper, carbon felt, Nafion membranes, Pt/C catalysts, etc. The specific scale is shown in Table S4. t_1 represents the time it takes to produce 2000m^3 of electrolyte. U_2 represents the cost of electrodes and membranes consumed to produce 2000m^3 of electrolyte Therefore, the consumption cost of producing 2000m^3 electrolyte is $U_2 = U_1 \times t_1/9000h$.

b) Assumption: Sulfuric acid in formic acid needs to be replaced for every 2000m^3 electrolyte produced.

c) The Table S5 shows the amount of raw materials required to produce 2000 m^3 of vanadium electrolyte.

d) Quoted from https://www.100ppi.com.

e) The electricity price is calculated at 1.2 ¥ kWh^{-1}

f) ¢ stand for the cent. 1 $=100 ¢

Table S6. Process cost of conventional method for producing 2000 m^3 $V^{3.5+}$ electrolyte (Production capacity: Industrial scale).

Component	Requirement[b)]	Unit cost	Cost ($)
**Reducing agent (SO_2)**	77.42 ton	501.52$ t^{-1} [c)]	38827.678
**Sulfuric acid**	6000 kmol	573.6¢ $kmol^{-1}$	34416
**Electricity consumption**	72.36 MWh	170$ MWh^{-1}	12301.2
**Consumable** [a)]	--	--	766980.858
	**Total**		852525.736

a) Assumptions: Electrode and membrane need to be replaced every 9000h operation. U_3 represents the total cost of electrode and membrane, including carbon felt, Titanium mesh, Nafion membrane, IrO_2 catalysts, etc. The specific scale is shown in Table S4. t_2 represents the time it takes to produce 2000m^3 of electrolyte. U_4 represents the cost of electrode and membrane consumed to produce 2000m^3 of electrolyte Therefore, the consumption cost of producing 2000m^3 electrolyte is $U_4 = U_3 \times t_2/9000h$.

b) The Table S6 shows the amount of raw materials required to produce 2000 m^3 of vanadium electrolyte

c) Quoted from http://china.makepolo.com.

Table S7. Performance comparison of vanadium electrolytes.

Current (mA/cm^2)	Coulomb efficiency	Energy efficiency	Cycle	Discharge capacity retention（%）	Reference
**100**	**94.1%**	**81.2%**	**120**	**81**	**This work**
80	95%	86%	200	34.5	Nat. Commun., 2019, 10, 4412
50	97.6%	89.2%	--	--	ACS Sustain. Chem. Eng., 2022, 10, 6181-6189
100	91%	65%	50	--	Electrochim. Acta, 2019， 309， 148-156
160	86%	67%	150	30	Electrochim. Acta, 2018， 259， 11-19
50	93%	86%	--	--	Electrochim. Acta, 2019， 303， 21-31
100	90%	78%	--	--	J． Power Sources， 2019， 415， 62-68
40	99%	74%	200	93	Electrochim. Acta, 2014， 121， 321-327
80	93%	85%	100	58.2	J． Power Sources， 2023， 555， 232330
50	90.2%	86.8%	100	83.3	ACS Sustainable Chem. Eng., 2022, 10, 9443–9452
60	93%	65%	150	--	J. Energy Chem., 2021, 57, 238-246
60	97.5%	83%	100	--	J． Power Sources， 2017， 355， 23-30
60	97%	80%	--	--	Energy Environ. Sci., 2012,5, 6299-6303

Reference

[1] J. Y. Heo, J. Y. Han, S. Kim, S. Yuk, C. Y. Choi, R. Kim, J. H. Lee, A. Klassen, S. K. Ryi, H. T. Kim, *Nat. Commun*. **2019**, 10, 4412;

[2] B. G. Kim, S. J. Lee(Daejeon KR), US9406961B2 2016; b) M. Skyllas-Kazacos, M. Rychick, R. Robins(Unisearch limited), AU.4786567 **1988**.

[3] M. Kazacos, I. Skyllas-Kazacos(VRB Power Systems, M. , US7078123B2 **2006**.
